# Facile Synthesis of Heterostructured WS_2_/Bi_2_MoO_6_ as High-Performance Visible-Light-Driven Photocatalysts

**DOI:** 10.1186/s11671-017-2157-y

**Published:** 2017-05-30

**Authors:** Jiyun Gao, Chenhui Liu, Fang Wang, Lijuan Jia, Kaijiao Duan, Tiancheng Liu

**Affiliations:** 1 Joint Research Centre for International Cross-border Ethnic Regions Biomass Clean Utilization in Yunnan, Kunming, 650500 China; 2 Education Department of Yunnan, Key Laboratory of Resource Clean Conversion in Ethnic Region, Kunming, 650500 China; 3 Key Laboratory of Comprehensive Utilization of Mineral Resources in Ethnic Regions, Kunming, 650500 China; 4 College of Chemistry and Environment, Yunnan MinZu University, Kunming, 650500 China

**Keywords:** WS_2_/Bi_2_MoO_6_, Solvothermal, Heterostructure, Visible-light driven, Photocatalysis

## Abstract

In this paper, novel WS_2_/Bi_2_MoO_6_ heterostructured photocatalysts were successfully fabricated via a facile solvothermal growth method using pre-exfoliated layered WS_2_ nanoslices as a substrate. The structure, morphology, and optical properties of the as-prepared WS_2_/Bi_2_MoO_6_ samples were characterized by XRD, XPS, SEM, TEM (HRTEM), and UV-vis diffuse reflectance spectra (DRS). Results confirmed the existence of an excellent nanojunction interface between layered WS_2_ nanoslices and Bi_2_MoO_6_ nanoflakes. Under visible light (>420 nm), the WS_2_/Bi_2_MoO_6_ composites exhibit significantly enhanced photocatalytic activity compared with pure Bi_2_MoO_6_ toward the decomposition of rhodamine B (RhB). Meanwhile, the active species trapping experiments indicated that holes (h^+^) were the main active species during the photocatalytic reaction. The enhanced photocatalytic performance can be ascribed to the effective light harvesting, fast photogenerated electron–hole pairs separation, and excellent charge carrier transport of the WS_2_/Bi_2_MoO_6_ heterostructures. Moreover, the prepared WS_2_/Bi_2_MoO_6_ composites also show good structural and activity stability in repeatability experiments.

## Background

The photocatalysis is widely regarded as one of the most promising environmental remediation technique due to the clean energy utilization method [1, 2]. Generally, some accepted that high-efficient photocatalysts with wide forbidden gap, such as TiO_2_ and ZnO, can only utilize ultraviolet light irradiation [3]. As to practical application, photocatalysis strategy will be a huge boost once a photocatalyst can favorably absorb the abundant solar energy in visible region. For this purpose, many attempts to probe visible-light photocatalyst for sufficient solar energy utilization by using the narrow band semiconductor [4–6]. Despite the single-phase photocatalyst can be excited smoothly by visible light, it still manifests low energy conversion efficiency due to poor charge separation efficiency resulting from rapid recombination of photo-induced electrons and holes [7]. It is widely accepted that the heterostructure can improve the separation probability of light-induced charge because the contact interfacial region of heterojunction will provide an internal electric field to restrain the recombination probability, thus resulting in an efficient photocatalytic performance. In general, the designed heterostructure will adopt at least one narrow band semiconductor to harvest more visual-light energy and then to generate more photo-induced charges [8, 9].

As a novel photocatalyst, Bi_2_MoO_6_ has received attention in the field of visual-light-driven photocatalysis because it possesses distinct sandwiched layered structure [10, 11]. As previously mentioned, the pure Bi_2_MoO_6_ is not suitable for the utilization as an efficient visible-light photocatalyst due to the high recombination probability of photogenerated charge carrier. Therefore, some effective strategies to meet this challenge by using the architecture of proper hybrid nanostructure and especially the introduction of two dimensional (2D) nanosheets have been proved as an effectual approach to strengthen interfacial charge transfer between two components in the process of photocatalytic reaction. Obviously, it is anticipated that the heterostructure between Bi_2_MoO_6_ and 2D layered material will increase photocatalytic efficiency by visual-light irradiation [8].

Layered transition metal dichalcogenides (TMDs) are widely regarded as a kind of promising loading material because of their analogous graphene reticular structure [12, 13]. Especially, monolayer and few layers of TMDs have important application for catalysis and energy storage due to their distinct electronic properties and high specific surface areas [14, 15]. For example, monolayered and few-layer MoS_2_ have recently paid the attention of the scientific community in photocatalysis research, which ascribes the lack of interlayer coupling and the absence of inversion symmetry resulting in the photoelectric property that differ markedly from those of the bulk [14, 16, 17]. From the material design perspective for an efficient visible-light-driven sensitized heterojunctional photocatalyst, the primary concern is that the hybrid narrow band gaps (1.1–1.7 eV) can closely match the solar spectrum [18]. In fact, the typical 2D layered semiconductors, such as MoS_2_ or g-C_3_N_4_, have received significant attention to explore potential photocatalysis applications, which lead to TMD nanosheet which is often utilized as a supporter to establish the heterostructured composite photocatalysts via different energy band hybrid strategies [19, 20]. For instance, the hierarchical MoS_2_/Bi_2_MoO_6_ composites exhibited an efficient performance for photocatalytic oxidation of rhodamine B under visible-light irradiation [21]. However, the mono- or few-layer heterostructured architecture of WS_2_/Bi_2_MoO_6_ as a visual-light photocatalyst has not been reported.

Herein, we demonstrated a facile strategy to fabricate heterostructured WS_2_/Bi_2_MoO_6_ composite via a facile solvothermal growth method using pre-exfoliated layered WS_2_ nanoslices as a supporter. The WS_2_/Bi_2_MoO_6_ exhibits excellent photocatalytic activity towards the degradation of rhodamine B (RhB) under visible-light (*λ* > 420 nm) irradiation. According to the microstructure characterization analysis of XRD, XPS, SEM, and TEM, the possible photocatalytic mechanism of the few-layer WS_2_/Bi_2_MoO_6_ composite was also elucidated. It is believed that the formation of junctions between Bi_2_MoO_6_ and WS_2_ can allow the prompt migration of photogenerated charge and reduce the self-agglomeration. It is postulated that the excellent photocatalytic activity of WS_2_/Bi_2_MoO_6_ should be ascribed to its high migration efficiency of photo-induced carriers and the interfacial electronic interaction. These results also probably provide a valuable perspective to insight into the design of other heterostructured photocatalysts.

## Methods

### Preparation of the Few-Layer WS_2_ Nanoslices

The liquid exfoliation of layered commercial WS_2_ was accomplished following the modified report method [22]. Briefly, 50 mg commercial WS_2_ powder (purchased from Aladdin Industrial Corporation) was added to 20 mL of ethanol/water with EtOH volume fractions of 40% added as dispersion solvent. The sealed flask was sonicated for 10 h, and then the dispersion was centrifuged at 3000 rpm for 20 min to remove aggregations. Finally, the supernatant was collected to obtain few-layer WS_2_ nanoslices. To determine the concentrations of 2D nanosheets in the supernatant, we estimated the mass remaining in the supernatant by measuring the UV-vis absorption spectrum at fixed wavelength of 630 nm. The calculation result by virtue of Lambert–Beer Law indicated that the exfoliated WS_2_ dispersion concentration was about 0.265 ± 0.02 mg/ml.

### Synthesis of Hierarchical WS_2_/Bi_2_MoO_6_ Composites

The WS_2_/Bi_2_MoO_6_ samples were synthesized using a facile solvothermal method. Typically, 2 mmol of Bi(NO_3_)_3_·5H_2_O was added to 10 mL of ethylene glycol solution containing dissolved Na_2_MoO_4_·2H_2_O with the Bi/Mo molar ratio of 2:1 under magnetic stirring. An appropriate amount of exfoliated WS_2_ nanoslices was dispersed into 20 mL ethanol and ultrasonicated at room temperature for 45 min. Then, it was slowly added into the above solution, followed by stirring for 10 min to form a homogeneous phase. The resulting solution was transferred into a 50-mL Teflon-lined stainless steel autoclave and kept at 160 °C for 10 h. Subsequently, the autoclave was cooled to room temperature gradually. Finally, the precipitate was centrifuged and washed with ethanol and deionized water several times and dried in a vacuum oven at 80 °C for 6 h. According to this method, WS_2_/Bi_2_MoO_6_ composites with different WS_2_ mass ratios (1, 3, 5, and 7 wt%) were synthesized. For comparison, the blank Bi_2_MoO_6_ was prepared in the absence of WS_2_ using the same experimental conditions.

### Characterization of Photocatalysts

Structure and morphology of the sample was investigated by scanning electron microscopy (SEM; JEOL JSM-6701F, Japan), transmission electron microscopy (TEM; JEOL 2100, Japan), high-resolution transmission electron microscopy (HRTEM; JEOL 2100, Japan), and powder X-ray diffraction (XRD; Bruker D8 Advance using Cu-Kα radiation source, *λ* = 1.5406 Å, USA). The ultraviolet-visible diffuse reflectance spectra (DRS) of samples were performed at room temperature in the range of 200–800 nm on a UV-vis spectrophotometer (Cary 500 Scan Spectrophotometers,Varian, USA) equipped with an integrating sphere attachment. The electronic states of surface elements of the catalysts were identified using X-ray photoelectron spectroscopy (XPS; Shimadzu Corporation, Japan, Al-Kα X-ray source).

### Measurement of Photocatalytic Activity

In all catalytic activity of experiments, 50 mg of the samples were added to aqueous RhB solution (50 mL, 10 mg/L) magnetically stirred in a Pyrex glass vessel and then radial irradiated with a 300 W Xe arc lamp (PLS-SXE 300, Beijing Perfect Company, Labsolar-III AG) to provide visible light with *λ* ≥ 420 nm by an ultraviolet UVCUT-420 nm cut-off filter (CE Aulight. Inc). The distance between the ultraviolet filter and aqueous RhB solution was about 6.5 mm. And the power density of visible light was 150 mW/cm^2^, which was estimated by the optical power meter (PD130, Thorlabs, USA). Prior to irradiation, the suspension was kept in the dark under magnetically stirred for 30 min to ensure the establishing of an adsorption/desorption equilibrium. At given time intervals, 2 mL were collected from the suspension and immediately centrifuged; the concentration of RhB after illumination was monitored at 553 nm by using UV-vis spectrophotometer (Shimadzu UV-2550, Shimadzu Corporation, Japan). The relative concentrations (*C*/*C*_0_) of the RhB were determined by the absorbance (*A*/*A*_0_) at 553 nm. All experiments were carried out at least in duplicate. The reported values were within the experimental error range of ±2%. Combining with Lambert–Beer law, the photocatalytic degradation rate constant (*k*) of RhB was obtained using the following formula:

$$ \ln \left({C}_0/ C\right)\kern0.5em =\kern0.5em  k t $$

where *C* is RhB concentration at reaction time *t*, *C*_0_ is the adsorption/desorption equilibrium concentration of RhB at the starting reaction time, and *A* and *A*_0_ are the corresponding absorbance values.

In addition, to identify the active species generated during photocatalytic reactivity, various scavengers were added into the solution of RhB, including 2 mM isopropanol (IPA, a quencher of ·OH), 2 mM disodium ethylenediamine tetraacetic acid (EDTA; a quencher of h^+^), and 2 mM *p*-benzoquinone (BQ; a ·O_2_^−^ scavenger), and 40 mL/min N_2_ (an electron quencher). The comparative trials of photocatalytic degradation were performed under the same reaction conditions as those mentioned above.

## Results and Discussion

### Micostructure and Morphology Analysis

**Fig. 1:**
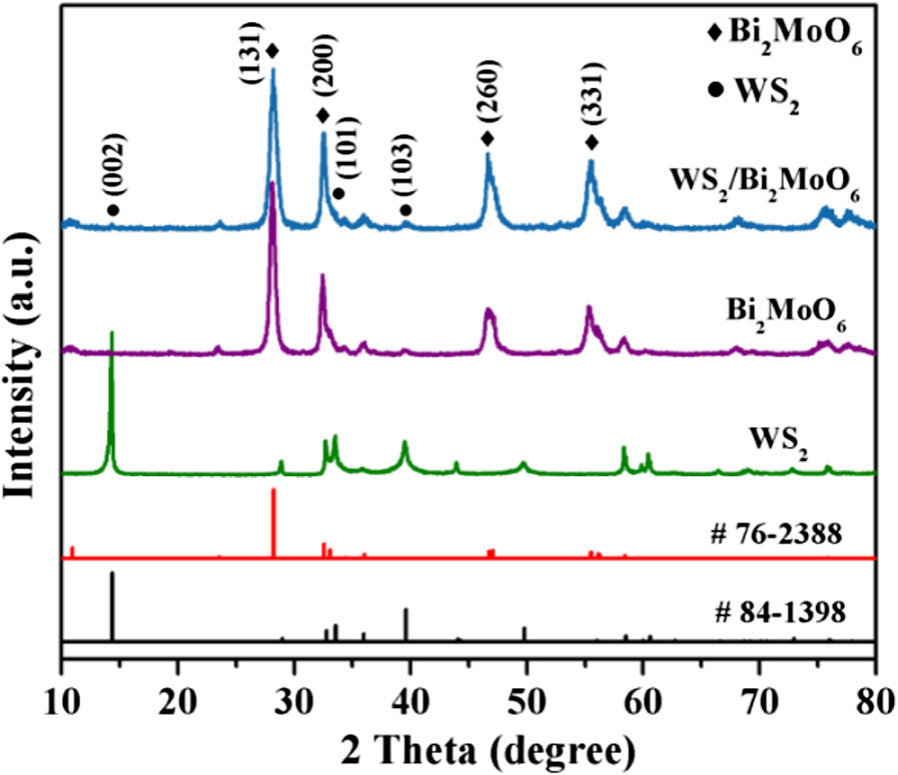
X-ray diffraction patterns of Bi_2_MoO_6_, few-layer WS_2_, and WS_2_/Bi_2_MoO_6_ (5 wt%) composite

In order to confirm the composition and crystal structure of the as-prepared samples, an XRD study was carried out. As shown in Fig. 1, it can be found that the pure WS_2_, five peaks located at 14.4°, 33.6°, 39.6°, 49.8°, and 58.5°, have been observed, which matched well with the (002), (101), (103), (105), and (110) crystal planes of WS_2_ (JCPDS card no. 84-1398). As for the pure Bi_2_MoO_6_, the diffraction peaks of (131), (200), (151), (260), (331), and (262) planes at 2*θ* = 28.2°, 32.5°, 36.0°, 47.1°, 55.4°,and 58.5°, which can be indexed to orthorhombic phase of Bi_2_MoO_6_ (JCPDS card no. 76-2388). In the case of the few-layer WS_2_/Bi_2_MoO_6_ composite materials, the XRD pattern only displays the characteristic diffraction peaks of hexagonal phase WS_2_ and orthorhombic phase Bi_2_MoO_6_. Furthermore, compared with the standard data for Bi_2_MoO_6_ (no. 76-2388), the existence of few-layer WS_2_ did not change the diffraction peak positions of Bi_2_MoO_6_ in the composite sample, indicating Bi_2_MoO_6_ nanoflakes grown on few-layer WS_2_ nanoslices rather than incorporated into the WS_2_ lattice. There is no trace of any impurity phase under the present resolution, which suggests the high purity of the as-prepared samples.

**Fig. 2:**
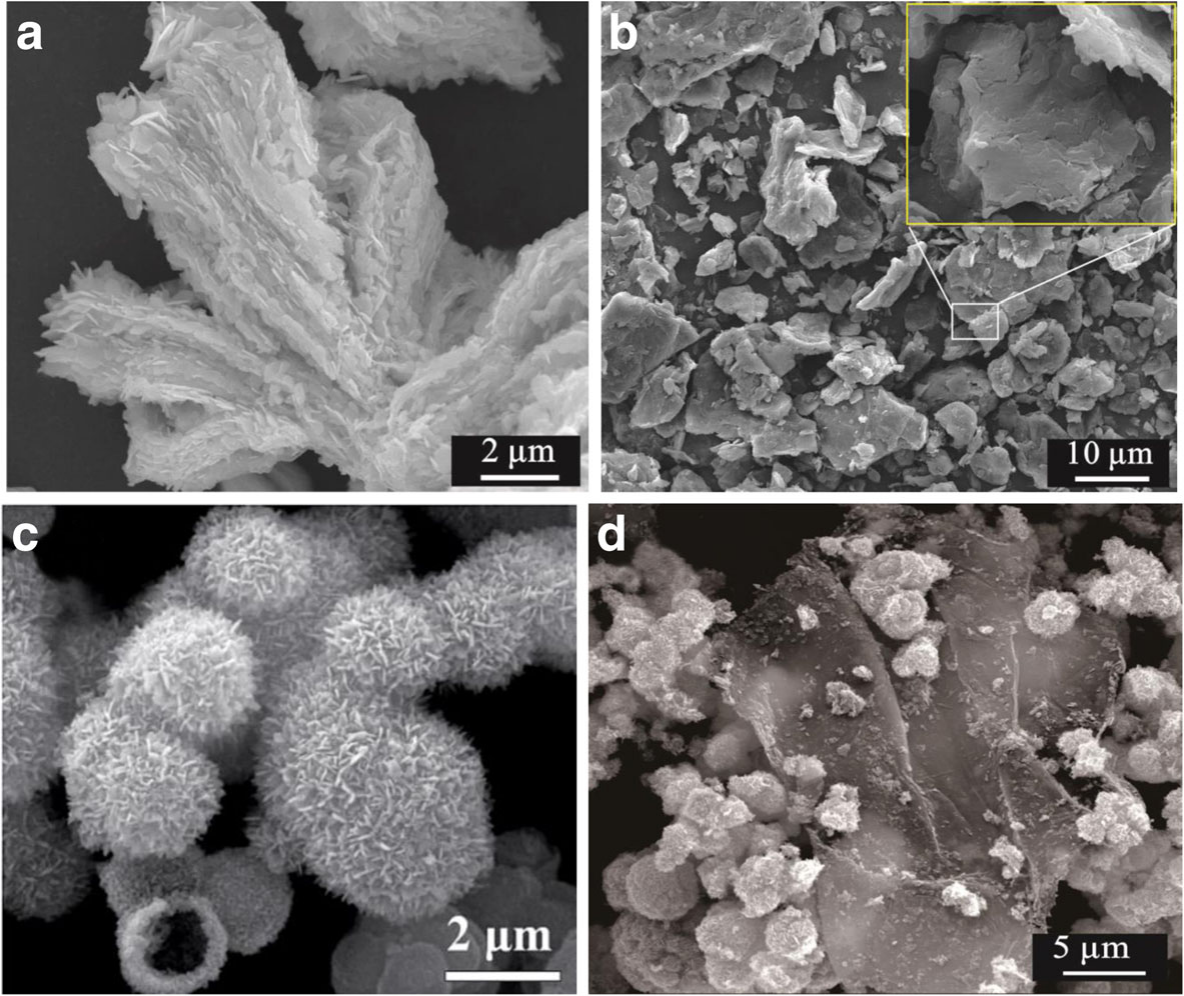
SEM images of the bulk raw WS_2_ (**a**), exfoliated WS_2_ nanoslices (**b**), pure Bi_2_MoO_6_ (**c**), and WS_2_/Bi_2_MoO_6_ (5 wt%) composite (**d**)

The morphologies of the as-synthesized samples were investigated using SEM. For comparison, SEM images of the bulk raw WS_2_ without sonicated treatment and exfoliated nanoslices are shown in Fig. 2a, b. The former displays a distinct multi-layer laminated morphology with about 20 μm in thickness, while the latter exhibits 2D sheet-like morphology with thickness varying from dozens of nanometers to 1–2 μm. The results demonstrate that the layered commercial WS_2_ have been stripped to few-layer WS_2_ nanoslices. Figure 2c shows the SEM image of pure Bi_2_MoO_6_. It can be seen that the Bi_2_MoO_6_ exhibited microsphere morphology with rough surfaces. Closer examination reveals that the microspheres consist of numerous secondary Bi_2_MoO_6_ nanoplates. Furthermore, when Bi_2_MoO_6_ was deposited onto the 2D few-layer WS_2_ via a facile solvothermal process (Fig. 2d), it can be clearly seen that the surfaces of WS_2_ nanoslices were uniformly covered by numerous two-dimensional Bi_2_MoO_6_ nanoplates (Fig. 2d) and that formed a WS_2_/Bi_2_MoO_6_ hierarchical structure.

**Fig. 3:**
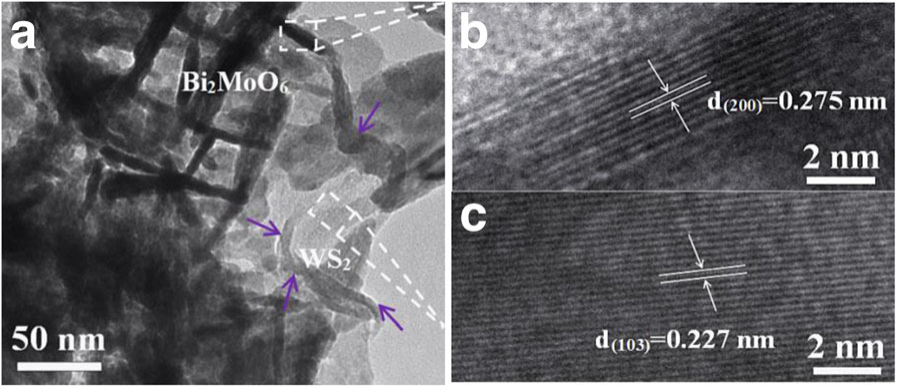
TEM (**a**) and HRTEM (**b**, **c**) images of WS_2_/Bi_2_MoO_6_ (5 wt%) composite

Further information about the nano-structure of the few-layer WS_2_/Bi_2_MoO_6_ composites was obtained from TEM (HRTEM) images. It is easy to observe in Fig. 3a that WS_2_ (purple arrows) shows a clear nanosheet structure which is similar to that of graphene, proving that graphene-like tungsten disulfide is obtained. Meanwhile, Bi_2_MoO_6_ nanoplates with diameters of about 50–100 nm were observed to grow on the WS_2_ nanosheets. HRTEM images (Fig. 3b, c) taken from Fig. 3a clearly display the resolved lattice fringes of 0.274 and 0.227 nm, which corresponds to the (200) planes of orthorhombic phase of Bi_2_MoO_6_ and the (103) planes of WS_2_, respectively. Therefore, the experimental results indicated that a coherent and tight heterojunction interface between few-layer WS_2_ and Bi_2_MoO_6_ was formed, which can benefit better charge separation and efficient electron transfer within the hybrid structure in comparison with pure Bi_2_MoO_6_.

### Electronic Structure and Spectrum Analysis

**Fig. 4:**
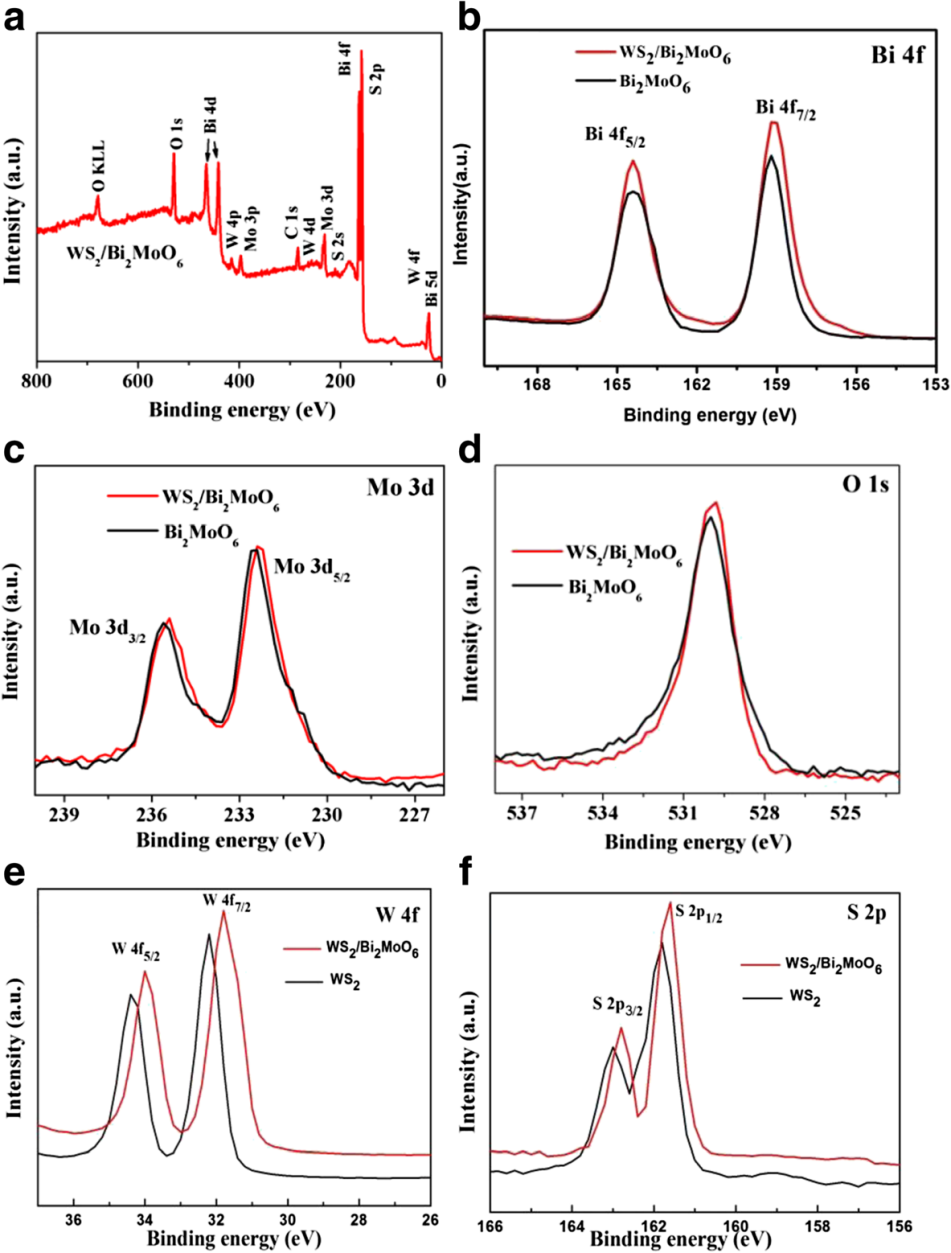
Survey XPS spectra of the WS_2_/Bi_2_MoO_6_ composite (**a**) and the high-resolution XPS spectra of Bi 4f (**b**), Mo 3d (**c**), O 1 s (**d**), W 4f (**e**), and S 2p (**f**) from Bi_2_MoO_6_, WS_2_, and the WS_2_/Bi_2_MoO_6_ composite (5 wt%)

The elemental composition and oxidation states of the fewlayer WS_2_/Bi_2_MoO_6_ composites were further determined by XPS spectra. Figure 4a shows the survey XPS spectra of the few-layer WS_2_/Bi_2_MoO_6_ (5 wt%) sample, which exhibits W, S, O, Bi, Mo, and C peaks. No peaks corresponding to other elements are observed. The peak for Bi 4f in the Bi_2_MoO_6_ (Fig. 4b) which appeared at 164.4 and 159.2 eV belonged to Bi 4f_5/2_ and Bi 4f_7/2_ of Bi^3+^ ions [23]. The Mo 3d binding energy (Fig. 4c) of 235.6 and 232.5 eV is consistent with the Mo 3d_3/2_ and Mo 3d_5/2_ of Mo^4+^ ions [23]. The asymmetric peaks of O 1 s (Fig. 4d) are located at 530.0 eV, which are characteristic of the Mo-O [24]. However, the binding energies of Bi 4f, Mo 3d, and O 1 s in the XPS spectra (Fig. 4b–d) of the hierarchical WS_2_/Bi_2_MoO_6_ slightly shift (about 0.2 eV) toward lower binding energies as compared with the pure Bi_2_MoO_6_. Meanwhile, in the hierarchical WS_2_/Bi_2_MoO_6_ composite, the values of W 4f_5/2_ (34.2 eV) and W 4f_7/2_ (32.0 eV) peaks (Fig. 4e) corresponding to WS_2_ are slightly lower (about 0.2 eV) than the pure WS_2_ (34.4 and 32.2 eV). Similarly, the high-resolution S 2p spectrum (Fig. 4f) also slightly shifts toward lower binding energies of 0.3 eV. These results could be ascribed to the strong interaction between WS_2_ and Bi_2_MoO_6_ resulting in an inner shift of Bi 4f, Mo 3d, O 1 s W 4f, and S 2p orbits [21, 25]. Therefore, by combining the XRD, SEM, TEM, and XPS investigations, it revealed that there are both WS_2_ and Bi_2_MoO_6_ species in the hierarchical WS_2_/Bi_2_MoO_6_ composite and that the heterojunctions are formed in their contact interface.

**Fig. 5:**
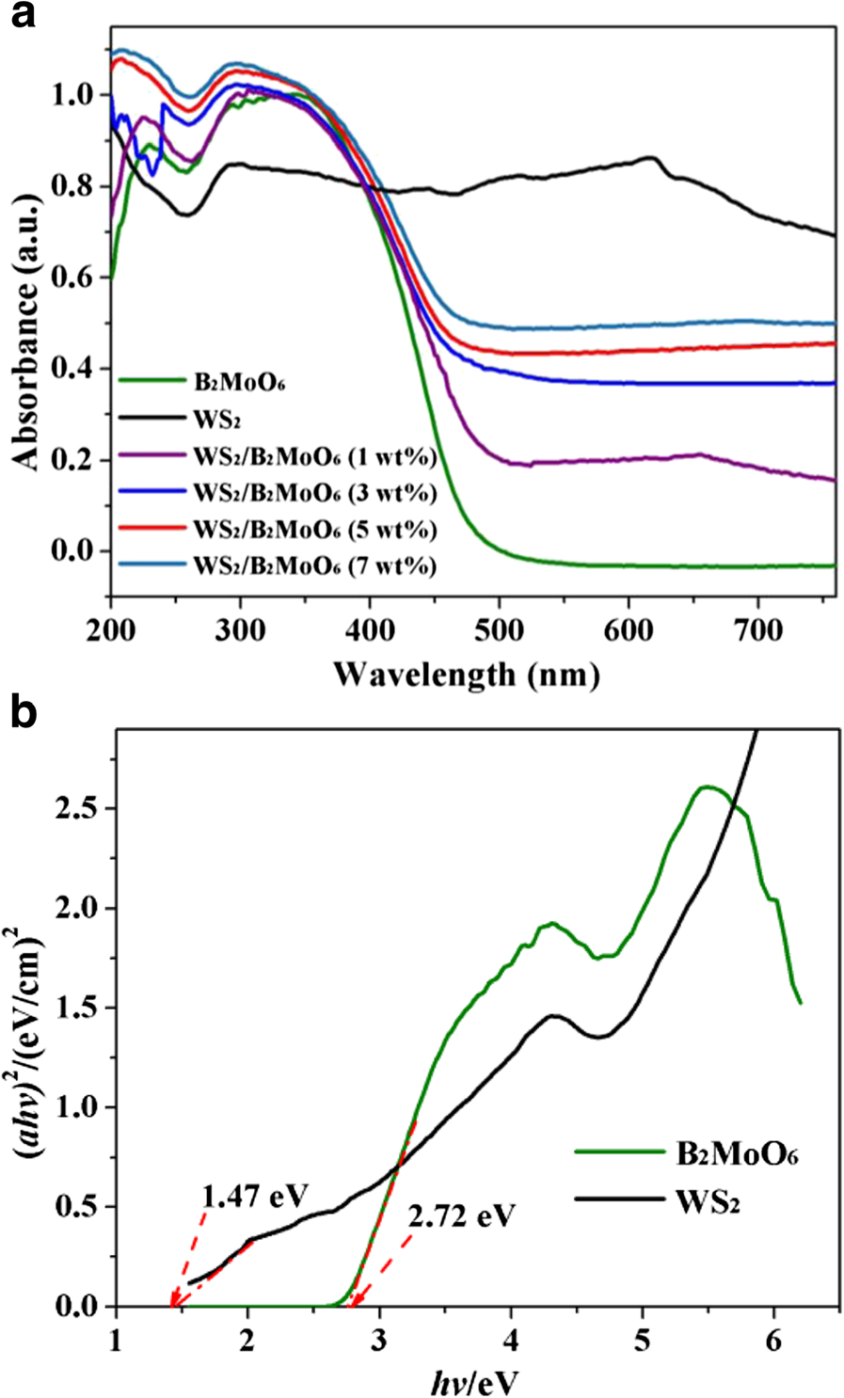
**a** UV-vis diffuse reflectance spectra (UV-Vis-DRS) of the as-prepared samples. **b** Plot of the transformed Kubelka–Munk functions vs. the energy of light

Figure 5a shows a comparison of the UV-vis diffuse reflectance spectra (UV-Vis-DRS) of the WS_2_, Bi_2_MoO_6_ and hierarchical WS_2_/Bi_2_MoO_6_ composite with different WS_2_ contents. It can be clearly seen that the absorption spectrum of pure Bi_2_MoO_6_ extends from the UV region to visible light at about 450 nm. When WS_2_ combined with Bi_2_MoO_6_, the absorption spectrum of the hierarchical composite exhibits an obviously redshift and more intensive absorption within the visible-light range in comparison with pure Bi_2_MoO_6_. Meanwhile, when the content of WS_2_ increased to a relatively high (3 to 7 wt%), the hierarchical composite display surprisingly strong absorption around 450–800 nm. These results clearly indicate that the composite photocatalyst could absorb more photons during photocatalytic reaction. Therefore, it can be revealed that the addition of WS_2_ nanoslices is beneficial for the visible-light absorbance of the WS_2_/Bi_2_MoO_6_ composite.

In addition, the optical band gap energies (*E*_g_) of samples were calculated by the following equation [26]:

$$ \alpha h v = A{\left( hv-{E}_g\right)}^{n/2} $$

where *α*, *hv*, *A*, and *E*_g_ are absorption coefficient, photon energy, proportionality constant, and bandgap, respectively. The value of *n* is determined by the type of transition (direct (*n* = 1) or indirect (*n* = 4)) [27, 28]. A plot of (*ahv*)^2^ versus (*hv*) is converted according the UV-Vis-DRS. As shown in Fig. 5b, the *E*_g_ values of pure WS_2_ and Bi_2_MoO_6_ have been estimated to be 1.47 and 2.72 eV, respectively.

### Photocatalytic Activity

**Fig. 6:**
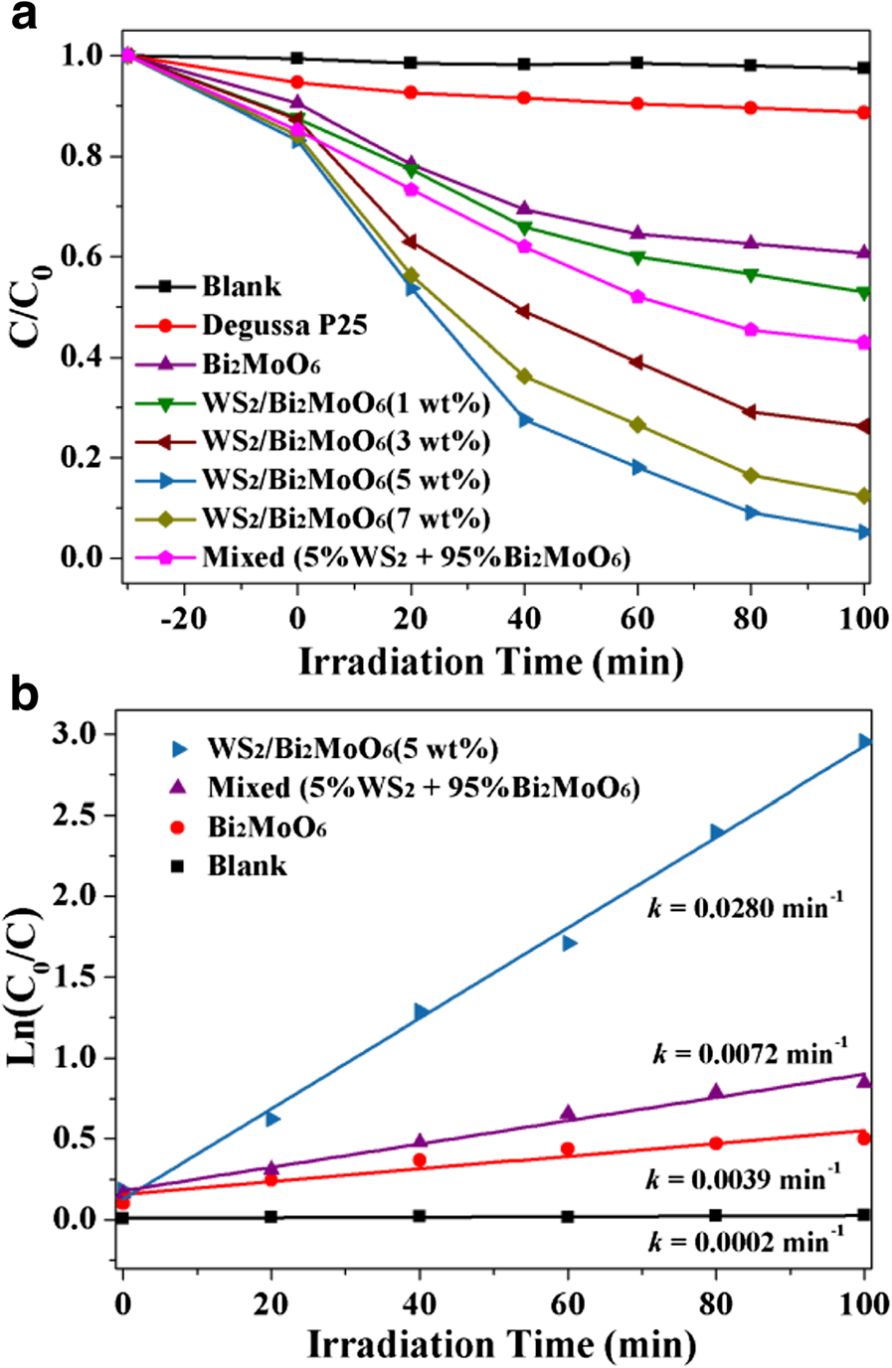
The photocatalytic activity (**a**) and kinetic fit (**b**) of the different photocatalysts for RhB degradation

The photocatalytic activities of the as-prepared samples were measured by degrading rhodamine B (RhB) under visible-light irradiation. For comparison, photocatalytic activities of pure Bi_2_MoO_6_ and mechanically mixed samples (5% WS_2_ and 95% Bi_2_MoO_6_) have also been investigated. As shown in Fig. 6a, the self-degradation effect of RhB under visible-light irradiation could be ignored. It can be clearly seen that the photodegradation rate of RhB by the pure Bi_2_MoO_6_ was only ~39% after 100 min of visible-light irradiation. Obviously, all the hierarchical WS_2_/Bi_2_MoO_6_ composites show better photocatalytic performance than the pure Bi_2_MoO_6_. ~48, ~74, ~95, and ~88% of RhB were degraded using 1% WS_2_/Bi_2_MoO_6_, 3% WS_2_/Bi_2_MoO_6_, 5% WS_2_/Bi_2_MoO_6_, and 7% WS_2_/Bi_2_MoO_6_, respectively. The results indicate that the optimal WS_2_ content in WS_2_/Bi_2_MoO_6_ composite exists when the mass ratio is 5%. Meanwhile, it was noted that the WS_2_/Bi_2_MoO_6_ (5 wt%) composite exhibits remarkably superior photocatalytic activity than the mechanically mixed 5% WS_2_ and 95% Bi_2_MoO_6_. This strongly suggests that an effective nanojunction interface contact and strong interactions between WS_2_ and Bi_2_MoO_6_ are extremely useful to enhance the migration, transport, and separation processes of photogenerated carriers. Furthermore, such superior photocatalytic performances could be attributed to the good crystallization and high specific surface area of composites and the small sheet thickness of the WS_2_ substrate.

In addition, the pseudo-first-order kinetics model was used to fit the experimental data of the photocatalytic degradation of the RhB solution, and the results are given in Fig. 6b. The rate constant *k* is 0.0280 min^−1^ for the hierarchical WS_2_/Bi_2_MoO_6_ (5 wt%) composites, which is 3.8 and 7.1 times greater than those of mechanically mixed WS_2_ and Bi_2_MoO_6_ and pure Bi_2_MoO_6_, respectively. These results indicated that RhB could be degraded more efficiently by the hierarchical WS_2_/Bi_2_MoO_6_ composite photocatalyst.

**Fig. 7:**
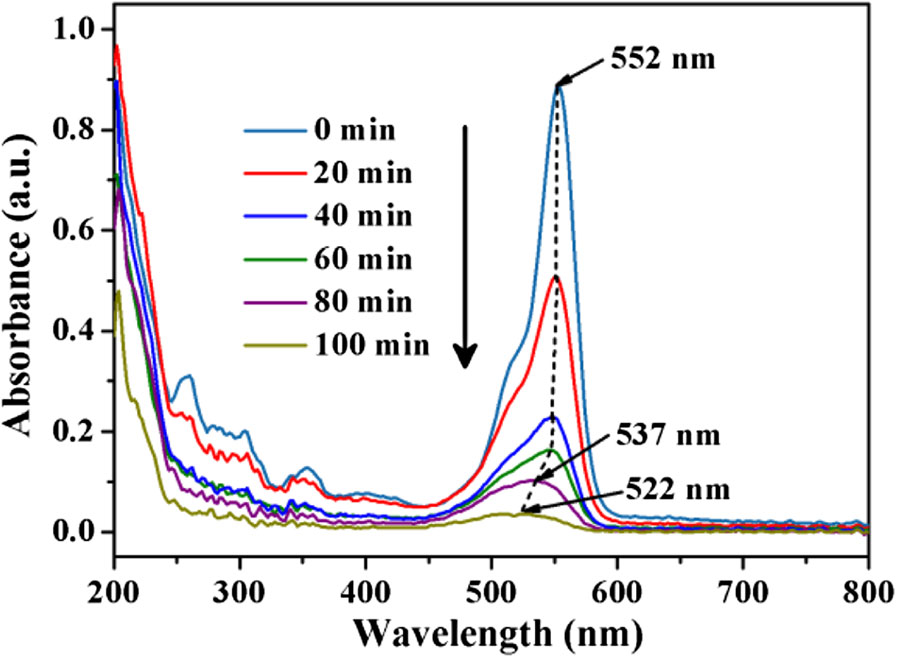
The optical adsorption spectra changes of RhB solution degradation over the WS_2_/Bi_2_MoO_6_ composite (5 wt%)

Figure 7 shows the UV-vis adsorption spectra changes of RhB solution degradation over the WS_2_/Bi_2_MoO_6_ (5 wt%) composite photocatalyst, which was performed to further study the photocatalytic degradation process of RhB. It can be seen that the main absorption peak of RhB gradually shifted from 552 to 537 nm, corresponding to the stepwise formation of a series of *N*-de-ethylated intermediates. As the visible-light irradiation process continues, the peak located at 537 nm is continued to shift and decrease, which indicates that the RhB molecules were further decomposed into smaller molecular fragments and the structure of RhB was also destroyed in the end. The two-step transition processes for photodegradation of RhB were also reported in several previous studies [29, 30]. Meanwhile, the suspension loses color gradually in the experiment, which further indicates that the structure of RhB has been destroyed in the end.

### Catalyst Stability

**Fig. 8:**
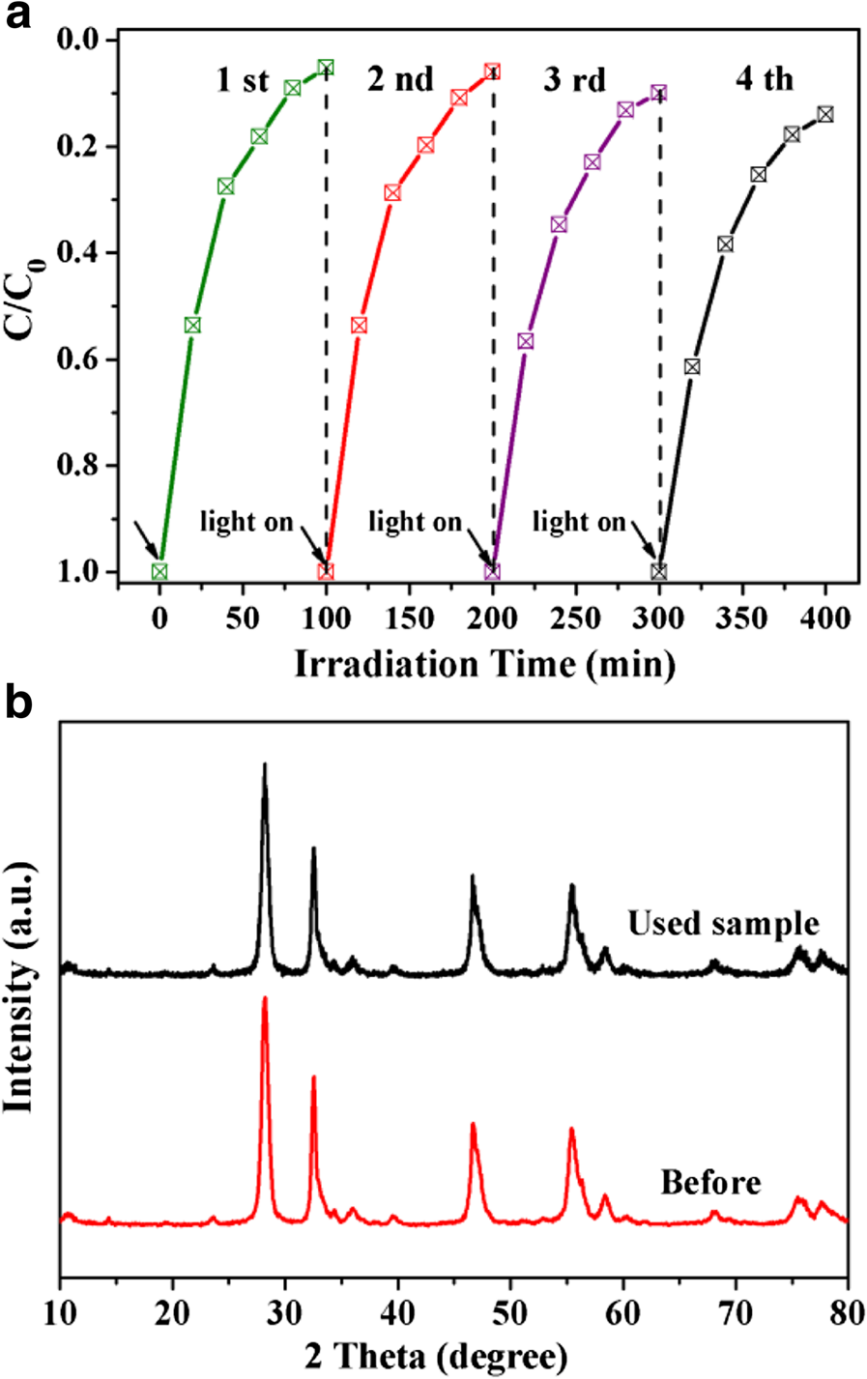
**a** Cycling runs for degradation of RhB over the WS_2_/Bi_2_MoO_6_ composite (5 wt%) under visible-light irradiation. **b** XRD patterns of the the WS_2_/Bi_2_MoO_6_ sample before and after four-cycle experiments

The photocatalytic stability of the hierarchical WS_2_/Bi_2_MoO_6_ composites was investigated by repeatability experiments for degradation of RhB, as shown in Fig. 8a. It can be found that the photocatalytic activity of WS_2_/Bi_2_MoO_6_ remains stable in the first two-cycle experiments. After four recycles, the catalysts did not show an obvious decrease in photocatalytic activity, demonstrating that WS_2_/Bi_2_MoO_6_ composite retained a relatively high degradation activity during the photodegradation process. Furthermore, the catalyst samples collected after four cycles was characterized by XRD measurement (Fig. 8b). It can be seen that the crystal structure and phase composition of WS_2_/Bi_2_MoO_6_ composite do not change after four photocatalytic reactions. Thus, the good structural stability ensures the WS_2_/Bi_2_MoO_6_ composite efficient photocatalysts working under visible-light irradiation.

### Possible Photocatalytic Mechanism

**Fig. 9:**
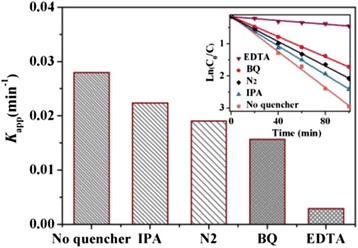
Rate constant *k*_app_ of the WS_2_/Bi_2_MoO_6_ (5 wt%) composite for the degradation of RhB in the presence of different scavengers under visible-light irradiation

Figure 9 shows the trapping experiment of main active species in the photocatalytic process of the WS_2_/Bi_2_MoO_6_ composite. Isopropanol (IPA), 1,4-benzoquinone (BQ), and disodium ethylenediamine tetraacetic acid (EDTA) acted as the scavengers for ·OH, ·O_2_^−^, and h^+^, respectively. It can be observed that the addition of 2 mM IPA or BQ in the RhB solution had a little effect on the rate constant *k*_app_, suggesting that ·OH and ·O_2_^−^ are the secondary active species during the photocatalytic reaction, not the main active species during the photocatalytic reaction. On the contrary, the *k*_app_ for degradation of RhB obviously decreased after the addition of 2 mM EDTA. Therefore, it can be confirmed that h^+^ play a key role for degradation of RhB. Furthermore, N_2_ was bubbled into the RhB solution at the rate of 40 mL/min to ensure that the reaction was operated without O_2_ as an electron quencher. The degradation of RhB showed a slight decrease in comparison with the case of the air-equilibrated solution and further indicated that ·O_2_^−^ played a minor role.

To explain the enhanced photocatalytic performance, conduction band (CB) and valence band (VB) of WS_2_ and Bi_2_MoO_6_ potentials should be calculated. For a semiconductor, the bottom CB and top VB can be estimated by the empirical formula [31]: *E*_CB_ = *X* − *E*_0_ − 0.5*E*_g_ and *E*_VB_ = *E*_CB_ + *E*_g_, where *E*_CB_ (*E*_VB_) is the CB (VB) edge potential; *X* is the electronegativity of the semiconductor; *E*_0_ is the energy of free electrons of the hydrogenscale (~4.5 eV *vs* NHE); and *E*_g_ is the band gap energy of the semiconductor obtained from the UV-visible diffuse reflectance absorption. The *X* values for WS_2_ and Bi_2_MoO_6_ are calculated to be 5.66 and 5.55 eV, respectively [28, 32, 33]. Thus, *E*_*C*B_ and *E*_VB_ values of WS_2_ are determined to be +0.43 and +1.9 eV and Bi_2_MoO_6_ are −0.31 and +2.41 eV, respectively.

**Fig. 10:**
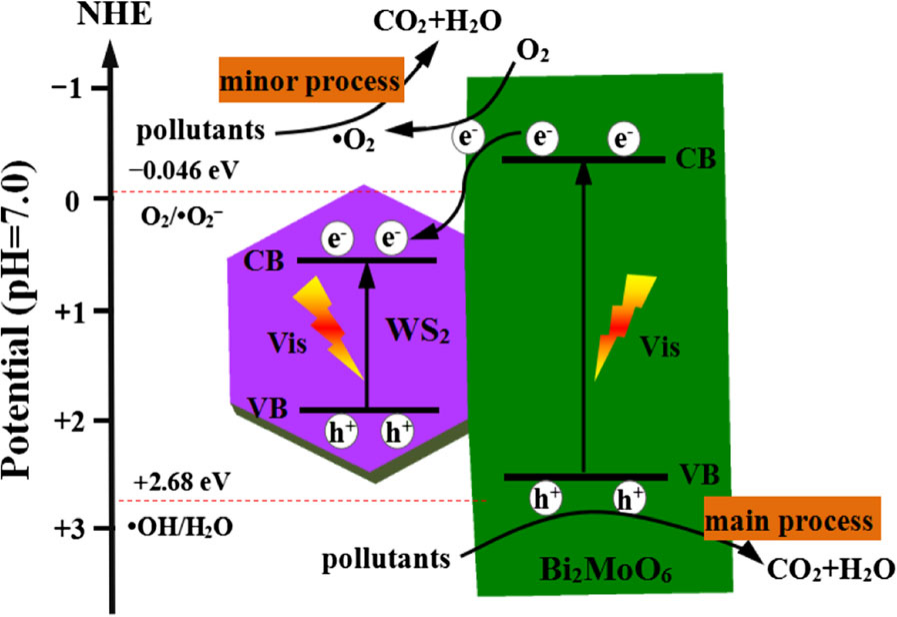
The proposed photocatalytic mechanism scheme of WS_2_/Bi_2_MoO_6_ composite under visible light (>420 nm)

On the basis of the above results, a possible photocatalytic mechanism scheme of the WS_2_/Bi_2_MoO_6_ composite photocatalyst is shown in Fig. 10. It can be found that WS_2_ and Bi_2_MoO_6_ are excited under visible-light irradiation and generate electrons and holes in their CB and VB, respectively. The electrons on CB of Bi_2_MoO_6_ will easily transfer WS_2_ due to the CB potential of Bi_2_MoO_6_ (−0.31 eV) is more negative than the CB potential of WS_2_ (0.43 eV) [29, 30]. The few-layer WS_2_ nanoslices could act as effective electron collectors, which was favorable to the separation of electron–hole pairs in Bi_2_MoO_6_. Therefore, this fast electron and hole transfer process can decrease the recombination of charges and prolong the lifetime of holes on VB of Bi_2_MoO_6_ [34]. The CB potential of WS_2_ (+0.43 eV) is more positive than E_0_ (O_2_/·O_2_^−^) (−0.046 eV) which suggests that the ·O_2_^−^ radicals were not formed through electrons reducing the dissolved O_2_ [35]. However, a few electrons on the CB of Bi_2_MoO_6_ can react with dissolved O_2_ to yield ·O_2_^−^ radicals because its potential (−0.31 eV) is more negative than E_0_ (O_2_/·O_2_^−^). Thus, the ·O_2_^−^ active species played a minor role. Meanwhile, the photo-induced holes on VB of Bi_2_MoO_6_ could not also directly oxidize the adsorbed H_2_O molecules to ·OH radicals because its potential (+2.41 eV) was lower than E_0_ (·OH/H_2_O) (+2.68 V) [36]. Finally, the main active species holes and minor active species ·O_2_^−^ act as a strong oxidizing agent to oxidize the organic pollutants (RhB) to CO_2_ and H_2_O. Therefore, the hierarchical WS_2_/Bi_2_MoO_6_ composites exhibit improved photocatalytic activity.

## Conclusions

In summary, a novel WS_2_/Bi_2_MoO_6_ heterostructured photocatalysts were successfully fabricated via a facile solvothermal growth method using pre-exfoliated layered WS_2_ nanoslices as a substrate. The hierarchical WS_2_/Bi_2_MoO_6_ exhibits excellent photocatalytic activity towards the degradation of rhodamine B (RhB) under visible-light irradiation. Based on the results of a series of structure and performance tests, it is believed that there formed a tight nanojunction interface between layered WS_2_ nanoslices and Bi_2_MoO_6_ nanoflakes, which make the photo-induced electrons be easily transferred to the WS_2_ substrate. As a result, the recombination of charges was decreased and the lifetime of holes was prolonged. Therefore, the hierarchical WS_2_/Bi_2_MoO_6_ composites exhibit much higher visible-light-driven photocatalytic activity than the pure Bi_2_MoO_6_. Furthermore, the WS_2_/Bi_2_MoO_6_ composites are very stable under visible-light irradiation and cycling photocatalytic tests. Thus, the as-prepared WS_2_/Bi_2_MoO_6_ photocatalyst has potential application for pollutant abatement.
